# Supplementary material for: Strain variation identifies a neural substrate for behavioral evolution in Drosophila
Source: bioRxiv. 2025 Aug 22:2025.08.15.670615. Preprint. [Version 2] doi: 10.1101/2025.08.15.670615 (PMC12393324; doi:10.1101/2025.08.15.670615)
Supplement: Supplement 11 [file NIHPP2025.08.15.670615v2-supplement-11.pdf]

bioRxiv preprint doi: https://doi.org/10.1101/2025.08.15.670615; this version posted August 22, 2025. The copyright holder for this preprint (which was not certified by peer review) is the author/funder, who has granted bioRxiv a license to display the preprint in perpetuity. It is made available under aCC-BY 4.0 International license.

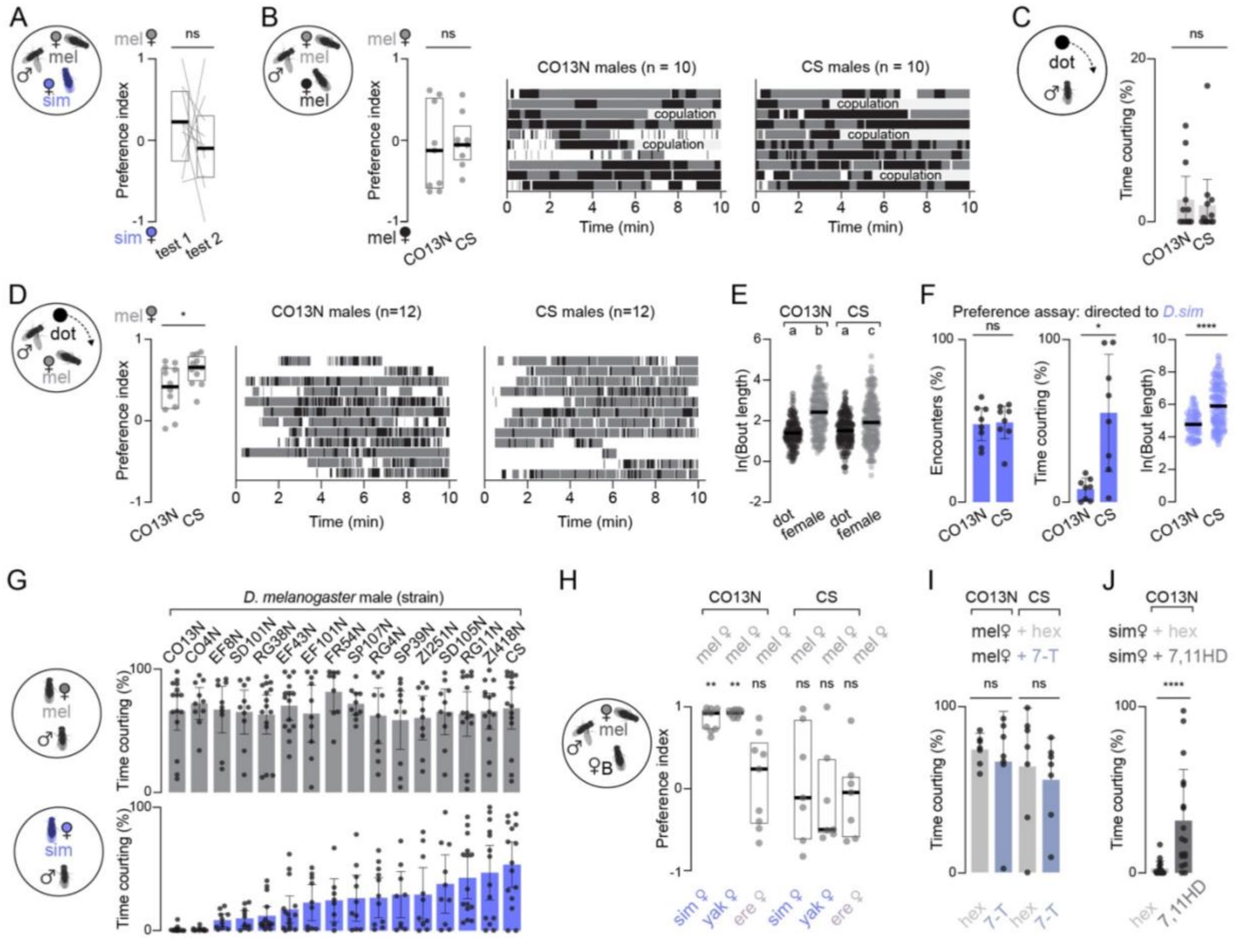

Figure S1. Male behavior implicates differential sensitivity to 7-T in establishing variation in strain preference. (**A**) Paired preference indices for CS males with a *D. melanogaster* and *D. simulans* female in two successive trials with individuals (lines), median (bold line), and inter-quartile range (IQR, box) shown. (**B**) Left: preference indices for CO13N and CS males paired with two equivalently reared *D. melanogaster* females with individuals (grey dots), median (bold line), and IQR (box) shown. Right: raster plots showing bouts of courtship over time toward each *D. melanogaster* female (grey or black) for CO13N and CS males, with copulations indicated. (**C**) Percent time spent courting a rotating dot projected onto the floor of the assay chamber (see Fig. 1D, Methods) for CO13N and CS males with individuals (grey dots), mean (bar), and 95% confidence interval (CI) shown. (**D**) Left: preference indices for CO13N and CS males paired with a *D. melanogaster* female (grey) and projected dot (black) with individuals (grey dots), median (bold line), and IQR (box) shown. Right: raster plots showing bouts of courtship over time toward the *D. melanogaster* female (grey) and the dot (black) for CO13N and CS males. (**E**) Natural log of bout lengths from (D) toward the dot and the female for CO13N and CS males with mean (bold line) shown. (**F**) Left: percent of all encounters (see Methods) with *D. simulans* female in the preference assay (Fig. 1C) for CO13N and CS males. Middle: percent time spent courting the *D. simulans* female for CO13N and CS males. Individuals (grey dots), mean (colored bars), and 95% CI (error bars) shown. Right: natural log of all bout lengths toward the *D. simulans* female in the preference assay (Fig. 1C) for CO13N and CS males with mean (bold line) shown. (**G**) Percent time spent courting a single *D. melanogaster* (grey, top) and *D. simulans* (blue, bottom) female for males of various strains with individuals (grey dots), means (colored bars), and 95% CI (error bars) shown. (**H**) Preference indices of CO13N and CS males paired with a *D. melanogaster* and a *D. simulans, D. yakuba,* or *D. erecta* female with individuals (grey dots), median (bold line), and IQR (box) shown. (**I**) Percent time that CO13N or CS males spent courting a single control *D. melanogaster* female treated with carrier solvent (hexane/hex, grey) or one perfumed with 7-T (blue-grey) with individuals

bioRxiv preprint doi: https://doi.org/10.1101/2025.08.15.670615; this version posted August 22, 2025. The copyright holder for this preprint (which was not certified by peer review) is the author/funder, who has granted bioRxiv a license to display the preprint in perpetuity. It is made available under aCC-BY 4.0 International license.

(grey dots), mean (colored bars), and 95% CI (error bars) shown. (**J**) Percent time CO13N males spent courting a single control *D. simulans* treated with hexane (hex, light grey) or one perfumed with 7,11-HD (dark grey) with individuals (grey dots), mean (bars), and 95% CI (error bars) shown. ns, $p > 0.05$; *, $p < 0.05$; **, $p < 0.01$; ****, $p < 0.0001$, groups that are not significantly different from one another ($p > 0.05$) after multiple comparisons indicated by shared letter codes. Details of statistical analyses and sample sizes are given in Table S1.

bioRxiv preprint doi: https://doi.org/10.1101/2025.08.15.670615; this version posted August 22, 2025. The copyright holder for this preprint (which was not certified by peer review) is the author/funder, who has granted bioRxiv a license to display the preprint in perpetuity. It is made available under aCC-BY 4.0 International license.

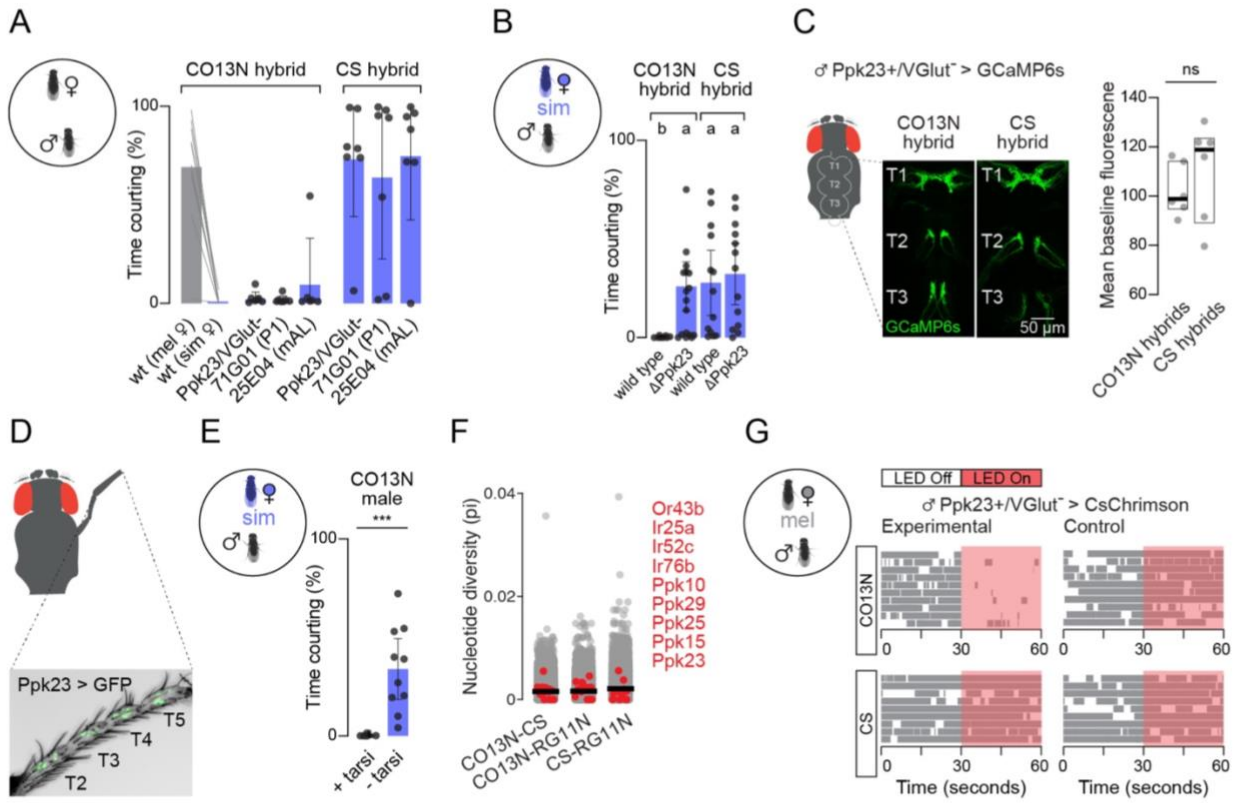

Figure S2. Cross-strain comparison of the sensory periphery. (**A**) Left: Paired courtship indices toward a *D. melanogaster* (grey) and *D. simulans* (blue) female for wild-type F1 hybrids generated by crossing CO13N and CS parents together (data where CO13N was the mother vs. where CS was the mother were not significantly different from one another and are combined in this figure). All other bars show courtship toward a *D. simulans* female for F1 crosses generated by crossing a wild-type selective CO13N or promiscuous CS female to the transgenic driver line indicated. (**B**) Percent time wild-type and ΔPpk23 null mutant CO13N and CS hybrid males spent courting a *D. simulans* female with means (bars), 95% confidence intervals (CI, error bars), and individuals (dots) shown. (**C**) Left: cartoon depicting the ventral nerve cord (VNC) of the male. Middle: 2-photon images of fluorescence from Ppk23-Gal4, VGlut-Gal80 > UAS-GCaMP6s in the VNC of selective CO13N (left) and promiscuous CS (right) hybrids. Right: mean baseline GCaMP6s fluorescence for drivers shown in (B). (**D**) Confocal image of a male foreleg with Ppk23-neurons marked by UAS-myr::GFP fluorescence. Tarsal segments are indicated. (**E**) Percent time that selective CO13N males courted a *D. simulans* female with intact forelegs or with foreleg tarsi (T2-T5) surgically removed. (**F**) Pairwise nucleotide diversity (pi) across all expressed transcripts (chemosensory gene transcripts highlighted in red and corresponding genes listed). Comparisons show sequenced RNA libraries generated from selective (CO13N) and promiscuous (CS, RG11N) or promiscuous (CS) and promiscuous (RG11N) foreleg samples. Mean pi indicated by black line. (**G**) Bouts of courtship toward a *D. melanogaster* female in the 30 seconds before and during optogenetic stimulus for Ppk23-Gal4, VGlut-Gal80 > UAS-CsChrimson CO13N and CS hybrid males shown in Fig. 2C raised on food with retinal (experimental) or without retinal (control).

bioRxiv preprint doi: https://doi.org/10.1101/2025.08.15.670615; this version posted August 22, 2025. The copyright holder for this preprint (which was not certified by peer review) is the author/funder, who has granted bioRxiv a license to display the preprint in perpetuity. It is made available under aCC-BY 4.0 International license.

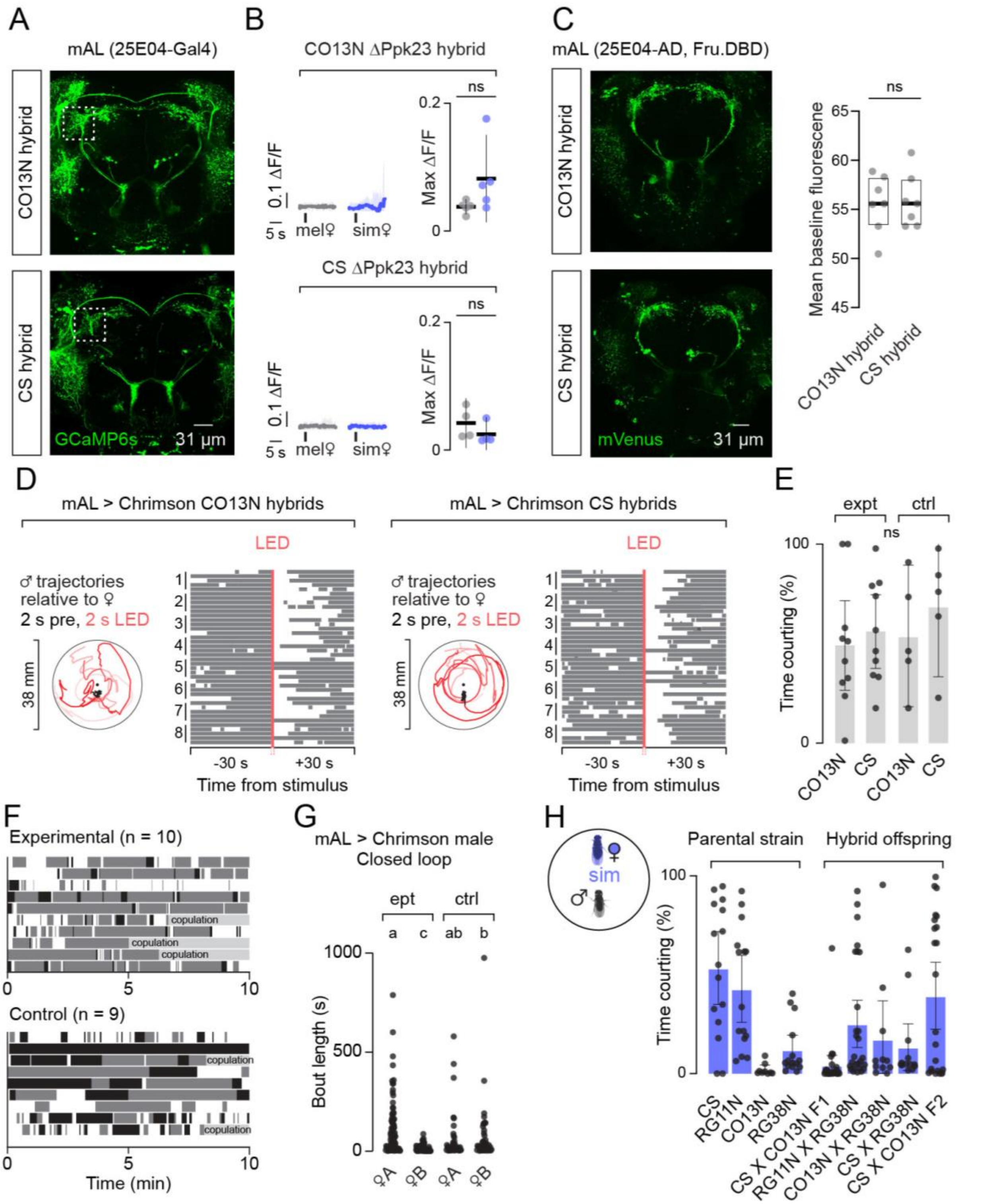

Figure S3. Cross-strain comparison of mAL neurons. (**A**) 2-P images of mAL neuron fluorescence in the brains of 25E04-Gal4 > UAS-GCaMP6s CO13N (top) and promiscuous CS (bottom) hybrids. White box indicates ROI for imaging mAL axons in the lateral protocerebral complex (LPC) shown in Fig. 4B. (**B**) mAL neuron responses in in ΔPpk23 mutant CO13N (top) and CS (bottom) hybrid males evoked by tapping the abdomen of a *D. melanogaster* (grey) or *D. simulans* (blue) female. Left panels: mean $\Delta F/F_0$ traces (thick line) aligned to tap (black tick) with 95% CI shading. Right panels: maximum responses showing individual data points (dots), mean (line), and 95% CI (error bars). (**C**) Left: 2-photon images of fluorescence from 25E04-p64.AD, Fru-Gal4.DBD >

bioRxiv preprint doi: https://doi.org/10.1101/2025.08.15.670615; this version posted August 22, 2025. The copyright holder for this preprint (which was not certified by peer review) is the author/funder, who has granted bioRxiv a license to display the preprint in perpetuity. It is made available under aCC-BY 4.0 International license.

UAS-GCaMP6s labeling mAL neurons in the brain of selective CO13N (top) and promiscuous CS (bottom) hybrids. Right: mean fluorescence from drivers shown in a standard ROI drawn over the LPC. (**D**) Left: Trajectories of representative mAL > UAS-CsChrimson CO13N (left) or CS (right) hybrid male in Fig. 4D plotted relative to the female's position in the 2 seconds prior to (black) and 2 seconds during optogenetic stimulus (red) for each of the five stimuli. Right: Courtship bouts 30 seconds before, during, and 30 seconds after mAL optogenetic activation for all males in in Fig. 4D**.** (**E**) Percent time that experimental and control CO13N and CS hybrids spent courting over the full optogenetic assay in Fig. 4G; individuals (dots), mean (grey bars), and 95% CI (error bars) shown. (**F**) Courtship bouts toward female "A" (grey) and female "B" (black) in the closed-loop experiment (Fig. 4H–J) across the full 10 min assay; copulations marked. (**H**) Percent time promiscuous (CS, RG11N) and selective (CO13N, RG38N) parental strains and hybrid crosses spent courting a *D. simulans* female with individuals (dots), mean (blue bars), and 95% CI (error bars) shown.

bioRxiv preprint doi: https://doi.org/10.1101/2025.08.15.670615; this version posted August 22, 2025. The copyright holder for this preprint (which was not certified by peer review) is the author/funder, who has granted bioRxiv a license to display the preprint in perpetuity. It is made available under aCC-BY 4.0 International license.

Table S1. Statistical analyses by figure panel. Provided as a separate Excel file containing details of statistical analyses performed for each applicable figure and supplementary figure.

Table S2. Fly genotypes and origin by figure panel. Associated genotypes of all males used in each figure section with their name as it appears in the text and their original source. *D. melanogaster* females were wild-type Canton-S. *D.simulans, D. erecta,* and *D. yakuba* females were wild-type.

Figure	Name	Male genotype	Source
1B	CO4N	wild-type	John Pool, UW Madison (see Pool et. al 2012, Lack et. al 2016)
	CO8N		
	CO13N		
	FR54N		
	FR126N		
	GU7		
	RG4N		
	RG11N		
	RG38N		
	SD101N		
	ZI418N		
	PC10	wild-type	Li Zhao, Rockefeller
	Canton-S	wild-type	Bloomington (#64349)
	Oregon-R	wild-type	Bloomington (#25211)
1C	CO13N	wild-type	
	Canton-S		
1E	CO13N	wild-type	
	Canton-S		
1F	CO13N	wild-type	
	RG38N		
	Canton-S		
	ZI418N		
1G	CO13N	wild-type	
	RG38N		
	Canton-S		
	ZI418N		
2A	CO13N hybrid, Ppk23^{+}/VGlut^{-} > GCaMP	CO13N (wt) X w; VGlut-Gal80; Ppk23-Gal4, UAS-GCaMP6s	Kristin Scott, UC Berkley (see Thistle et. al 2012), Bloomington (#58448, 42749)
	CS hybrid, Ppk23^{+}/VGlut^{-} > GCaMP	CS (wt) X w; VGlut-Gal80; Ppk23-Gal4, UAS-GCaMP6s	
2C-G	CO13N hybrid, Ppk23^{+}/VGlut^{-} > Chrimson	CO13N (wt) X w; VGlut-Gal80; Ppk23-Gal4, UAS-CsChrimson:mVenus	Kristin Scott, UC Berkley (see Thistle et. al 2012),

bioRxiv preprint doi: https://doi.org/10.1101/2025.08.15.670615; this version posted August 22, 2025. The copyright holder for this preprint (which was not certified by peer review) is the author/funder, who has granted bioRxiv a license to display the preprint in perpetuity. It is made available under aCC-BY 4.0 International license.

		Experimental (expt) flies raised on food with retinal; control (ctrl) flies raised on food without retinal	Bloomington (#58448, 55136)
	CS hybrid, Ppk23^{+}/VGlut^{-} > Chrimson	CS (wt) X w; VGlut-Gal80; Ppk23-Gal4, UAS-CsChrimson:mVenus<br>*Experimental (expt) flies raised on food with retinal; control (ctrl) flies raised on food without retinal*	
3A-B	CO13N hybrid, P1 > GCaMP	CO13N (wt) X w; UAS-GCaMP6s; R71G01-Gal4	Bloomington (#42746, 39599)
	CS hybrid, P1 > GCaMP	CS (wt) X w; UAS-GCaMP6s; R71G01-Gal4	
3C	CO13N hybrid, P1 > GCaMP Rdl RNAi-knockdown	CO13N (wt) X w; UAS-GCaMP6s; R71G01-Gal4, UAS-Rdl.RNAi(8-10)	Bloomington (#42746, 39599, 89903, see Liu et. Al 2009 for RNAi details)
	CO13N hybrid, P1 > GCaMP Gal4 control	CO13N (wt) X w; UAS-GCaMP6s; R71G01-Gal4	
3D	P1 > Rdl RNAi-knockdown	CO13N (wt) X w; +; R71G01-Gal4, UAS-Rdl.RNAi(8-10)	
	Gal4 control	CO13N (wt) X w; +; R71G01-Gal4	
4B	CO13N hybrid, mAL > GCaMP	CO13N (wt) X w; UAS-GCaMP6s; R25E04-Gal4	Bloomington (#42746, 49125)
	CS hybrid, mAL > GCaMP	CS (wt) X w; UAS-GCaMP6s; R25E04-Gal4	
4C	CO13N hybrid, mAL > Kir	CO13N (wt) X w; 25E04-p65.AD; Fru-Gal4.DBD, UAS-Kir2.1:tdTomato	Barry Dickson, Janelia (see Tirian and Dickson 2017), Bloomington (#68874, 600376, 9466)
	CO13N hybrid, control	CO13N (wt) X w; 25E04-p65.AD; Fru-Gal4.DBD	
	CS hybrid, mAL > NaChBac	CS (wt) X w; 25E04-p65.AD; Fru-Gal4.DBD, UAS-NaChBac:EGFP	
	CS hybrid, control	CS (wt) X w; 25E04-p65.AD; Fru-Gal4.DBD	
4D-H	CO13N hybrid, mAL > Chrimson	CO13N (wt) X w; 25E04-p65.AD; Fru-Gal4.DBD, UAS-CsChrimson:mVenus<br>*Experimental (expt) flies raised on food with retinal; control (ctrl) flies raised on food without retinal*	Barry Dickson, Janelia (see Tirian and Dickson 2017), Bloomington (#68874, 55136)
	CS hybrid, mAL > Chrimson	CS (wt) X w; 25E04-p65.AD; Fru-Gal4.DBD, UAS-CsChrimson:mVenus	

bioRxiv preprint doi: https://doi.org/10.1101/2025.08.15.670615; this version posted August 22, 2025. The copyright holder for this preprint (which was not certified by peer review) is the author/funder, who has granted bioRxiv a license to display the preprint in perpetuity. It is made available under aCC-BY 4.0 International license.

		Experimental (expt) flies raised on food with retinal; control (ctrl) flies raised on food without retinal	
S1A	Canton-S	wild-type	
S1B-F	CO13N	wild-type	
	Canton-S		
S1G	CO4N	wild-type	
	CO13N		
	EF8N		
	EF43N		
	EF101N		
	FR54N		
	RG4N		
	RG11N		
	RG38N		
	SD101N		
	SP39N		
	SP107N		
	ZI251N		
	ZI418N		
	Canton-S		
S1H-J	CO13N	wild-type	
	Canton-S		
S2A	CO13N hybrid	CO13N (wt) X CS (wt)	
	CO13N hybrid, Ppk23^{+}/VGlut^{-}	CO13N (wt) X w; Vglut-Gal80; Ppk23-Gal4	
	CO13N hybrid, P1	CO13N (wt) X w; +; R71G01-Gal4	
	CO13N hybrid, mAL	CO13N (wt) X w; +; R25E04-Gal4	
	CS hybrid, Ppk23^{+}/VGlut^{-}	CS (wt) X w; Vglut-Gal80; Ppk23-Gal4	
	CS hybrid, P1	CS (wt) X w; +; R71G01-Gal4	
	CS hybrid, mAL	CS (wt) X w; +; R25E04-Gal4	
S2B	CO13N wild-type	CO13N (wt) X CS (wt)	
	CO13N hybrid, ΔPpk23	ΔPpk23; +; + X CO13N (wt ♂)	
	CS hybrid	CS (wt)	
	CS hybrid, ΔPpk23	ΔPpk23; +; + X CS(wt ♂)	
S2C	CO13N hybrid, Ppk23^{+}/VGlut^{-} > GCaMP	CO13N (wt) X w; VGlut-Gal80; Ppk23-Gal4, UAS-GCaMP6s	

bioRxiv preprint doi: https://doi.org/10.1101/2025.08.15.670615; this version posted August 22, 2025. The copyright holder for this preprint (which was not certified by peer review) is the author/funder, who has granted bioRxiv a license to display the preprint in perpetuity. It is made available under aCC-BY 4.0 International license.

	CS hybrid, Ppk23$^+$/VGlut$^-$ > GCaMP	CS (wt) X w; VGlut-Gal80; Ppk23-Gal4, UAS-GCaMP6s	
S2D	Ppk23 > GFP	w; UAS-myr:GFP; Ppk23-Gal4	
S2E	CO13N	wild-type	
S2F	CO13N	wild-type	
	CS		
	RG11N		
S2G	CO13N hybrid, Ppk23$^+$/VGlut$^-$ > Chrimson	CO13N (wt) X w; VGlut-Gal80; Ppk23-Gal4, UAS-CsChrimson:mVenus *Experimental (expt) flies raised on food with retinal; control (ctrl) flies raised on food without retinal*	
	CS hybrid, Ppk23$^+$/VGlut$^-$ > Chrimson	CS (wt) X w; VGlut-Gal80; Ppk23-Gal4, UAS-CsChrimson:mVenus *Experimental (expt) flies raised on food with retinal; control (ctrl) flies raised on food without retinal*	
S3A	CO13N hybrid, mAL > GCaMP	CO13N (wt) X w; UAS-GCaMP6s; R25E04-Gal4	
	CS hybrid, mAL > GCaMP	CS (wt) X w; UAS-GCaMP6s; R25E04-Gal4	
S3B	CO13N hybrid, ΔPpk23 mAL > GCaMP	ΔPpk23; UAS-GCaMP6s; R25E04-Gal4 X CO13N (wt ♂)	
	CS hybrid, ΔPpk23 mAL > GCaMP	ΔPpk23; UAS-GCaMP6s; R25E04-Gal4 X CS (wt ♂)	
S3C	CO13N hybrid, mAL > GCaMP	CO13N (wt) X w; R25E04-p65.AD; Fru-Gal4.DBD, UAS-GCaMP6s	
	CS hybrid, mAL > GCaMP	CS (wt) X w; R25E04-p65.AD; Fru-Gal4.DBD, UAS-GCaMP6s	
S3D-G	CO13N hybrid, mAL > Chrimson	CO13N (wt) X w; 25E04-p65.AD; Fru-Gal4.DBD, UAS-CsChrimson:mVenus *Experimental (expt) flies raised on food with retinal; control (ctrl) flies raised on food without retinal*	
	CS hybrid, mAL > Chrimson	CS (wt) X w; 25E04-p65.AD; Fru-Gal4.DBD, UAS-CsChrimson:mVenus *Experimental (expt) flies raised on food with retinal; control*	

bioRxiv preprint doi: https://doi.org/10.1101/2025.08.15.670615; this version posted August 22, 2025. The copyright holder for this preprint (which was not certified by peer review) is the author/funder, who has granted bioRxiv a license to display the preprint in perpetuity. It is made available under aCC-BY 4.0 International license.

<table>
<tr><td></td><td></td><td>(ctrl) flies raised on food without retinal</td><td></td></tr>
<tr><td rowspan="9">S3H</td><td>CS</td><td rowspan="4">wild-type</td><td rowspan="9"></td></tr>
<tr><td>RG11N</td></tr>
<tr><td>CO13N</td></tr>
<tr><td>RG38N</td></tr>
<tr><td>CS X CO13N F1</td><td>CO13N (wt) X CS (wt)</td></tr>
<tr><td>RG11N X RG38N</td><td>RG38N (wt) X RG11N (wt)</td></tr>
<tr><td>CO13N X RG38N</td><td>CO13N (wt) X RG38N (wt)</td></tr>
<tr><td>CS X RG38N</td><td>RG38N (wt) X CS (wt)</td></tr>
<tr><td>CS X CO13N F2</td><td>CO13N/CS F1 hybrid X CO13N/CS F1 hybrid</td></tr>
</table>

bioRxiv preprint doi: https://doi.org/10.1101/2025.08.15.670615; this version posted August 22, 2025. The copyright holder for this preprint (which was not certified by peer review) is the author/funder, who has granted bioRxiv a license to display the preprint in perpetuity. It is made available under aCC-BY 4.0 International license.

Movie S1. Behavior of a selective (CO13N) *D. melanogaster* male in preference assay, offered a conspecific and *D. simulans* female. Representative video from a preference assay with identities of the participants indicated at the beginning of the clip and on subsequent frozen frames to aid with visual interpretation. Displayed at 2X speed.

Movie S2. Behavior of a promiscuous (CS) *D. melanogaster* male offered a conspecific and D. simulans female. Representative video from a preference assay with identities of the participants indicated at the beginning of the clip and on subsequent frozen frames to aid with visual interpretation. Videos are shown at 2X speed.

Movie S3. Behavior of a selective (CO13N) male toward a projected dot alone and when paired with a conspecific female. Sequential labeled video clips from assays in which a CO13N male interacts with a 3 mm dot projected onto the floor of the chamber alone and in the presence of a conspecific female. Videos are shown at 2X speed.

Movie S4. Behavior of a promiscuous (CS) male toward a projected dot alone and when paired with a conspecific female. Sequential labeled video clips from assays in which a CS male interacts with a 3 mm dot projected onto the floor of the chamber alone and in the presence of a conspecific female. Videos are shown at 2X speed.

Movie S5. Optogenetic activation of 7T-sensing neurons in a selective hybrid male. Representative video clips of CO13N hybrid males expressing CsChrimson in Ppk23^{+}/VGlut^{-} sensory neurons, reared either on food with retinal (experimental) or without retinal (control). In all clips, males court a conspecific female and are then exposed to a 2 second red LED stimulus (LED-on indicated by a red circle in the upper left corner). Videos are shown at 2X speed.

Movie S6. Optogenetic activation of 7T sensing neurons in a promiscuous hybrid male. Representative video clips of CS hybrid males expressing CsChrimson in Ppk23^{+}/VGlut^{-} sensory neurons, reared either on food with retinal (experimental) or without retinal (control). In all clips, males court a conspecific female and are then exposed to a 2 second red LED stimulus (LED-on indicated by a red circle in the upper left corner). Videos are shown at 2X speed.

Movie S7. Optogenetic activation of mAL neurons in a selective hybrid male paired with two conspecific females. Representative video clips of CO13N hybrid males expressing CsChrimson in mAL neurons (defined by the intersection of 25E04-p65.AD/Fru-Gal4.DBD), reared either on food with retinal (experimental) or without retinal (control). In all clips, males are paired with two conspecific females and exposed to a 2 second red LED stimulus (LED-on indicated by a red circle in the upper left corner). Videos are shown at 2X speed.

Movie S8. Optogenetic activation of mAL neurons in a promiscuous hybrid male paired with two conspecific females. Representative video clips of CS hybrid males expressing CsChrimson in mAL neurons (defined by the intersection of 25E04-p65.AD/Fru-Gal4.DBD), reared either on food with retinal

bioRxiv preprint doi: https://doi.org/10.1101/2025.08.15.670615; this version posted August 22, 2025. The copyright holder for this preprint (which was not certified by peer review) is the author/funder, who has granted bioRxiv a license to display the preprint in perpetuity. It is made available under aCC-BY 4.0 International license.

(experimental) or without retinal (control). In all clips, males are paired with two conspecific females and exposed to a 2 second red LED stimulus (LED-on indicated by a red circle in the upper left corner). Videos are shown at 2X speed.

Movie S9. Closed-loop ontogenetic activation of mAL neurons in a male paired with two conspecific females. Representative video clips of CS hybrid males expressing CsChrimson in mAL neurons (defined by the intersection of 25E04-p65.AD/Fru-Gal4.DBD), reared either on food with retinal (experimental) or without retinal (control). In all clips, males are paired with two conspecific females and exposed to 500 ms second red LED stimulus (LED-on indicated by a red circle in the upper left corner) whenever real-time tracking algorithm registers him < 2mm and oriented within a 60-degree arc of the arbitrarily chosen fictive "*D. simulans*" female. Automated tracking of head/thorax/abdomen and the identity (male, blue; female, gold; fictive *D. simulans,* green) overlaid on video. Videos are shown at 2X speed.
